# Cartilage Tissue Engineering Using Stem Cells and Bioprinting Technology—Barriers to Clinical Translation

**DOI:** 10.3389/fsurg.2018.00070

**Published:** 2018-11-27

**Authors:** Sam L. Francis, Claudia Di Bella, Gordon G. Wallace, Peter F. M. Choong

**Affiliations:** ^1^Department of Surgery, University of Melbourne, Melbourne, VIC, Australia; ^2^Department of Orthopaedics, St Vincent's Hospital, Melbourne, VIC, Australia; ^3^Biofab 3D, Aikenhead Centre for Medical Discovery, Melbourne, VIC, Australia; ^4^Australian Research Council, Centre of Excellence for Electromaterials Science, Intelligent Polymer Research Institute, University of Wollongong, Wollongong, NSW, Australia

**Keywords:** cartilage, stem cells, scaffolds, hydrogels, tissue engineering, bioprinting, bio fabrication

## Abstract

There is no long-term treatment strategy for young and active patients with cartilage defects. Early and effective joint preserving treatments in these patients are crucial in preventing the development of osteoarthritis. Tissue engineering over the past few decades has presented hope in overcoming the issues involved with current treatment strategies. Novel advances in 3D bioprinting technology have promoted more focus on efficient delivery of engineered tissue constructs. There have been promising *in-vitro* studies and several animal studies looking at 3D bioprinting of engineered cartilage tissue. However, to date there are still no human clinical trials using 3D printed engineered cartilage tissue. This review begins with discussion surrounding the difficulties with articular cartilage repair and the limitations of current clinical management options which have led to research in cartilage tissue engineering. Next, the major barriers in each of the 4 components of cartilage tissue engineering; cells, scaffolds, chemical, and physical stimulation will be reviewed. Strategies that may overcome these barriers will be discussed. Finally, we will discuss the barriers surrounding intraoperative delivery of engineered tissue constructs and possible solutions.

## Introduction

Articular cartilage defects pose a significant burden to patients both symptomatically and functionally, leading to reduced quality of life. Younger patients particularly have no long-term treatment strategy and face multiple operations and possible complications in their lifetime due to the inevitable development of osteoarthritis (1, 2).

Several surgical options exist in current practice (Discussed in detail under the current treatment strategies section); however, they only provide short-term benefit in certain subsets of patients based on the nature of the defect and host factors. Fibrocartilage production is the major barrier in long-term viability of these methods and is detrimental to joint function (3).

Treatment strategies currently being pursued in research, focus on the development of 3D bioprinting of engineered cartilage tissue. This is a diverse field with many factors warranting optimization and integration to deliver the ultimate 3D tissue structure. However, there are several obstacles with each individual component, collectively leading to a barrier with respect to clinical use of cartilage tissue engineering and 3D bioprinting for cartilage repair. These hurdles will need to be addressed prior to any design of a human clinical trial.

## Articular cartilage: structure and repair

Articular cartilage is primarily composed of hyaline tissue. Hyaline cartilage is a specialized tissue found in most joints and provides low friction and shock absorption. It also provides a structural and biological barrier between two bone surfaces leading to smooth uniform range of motion (4).

**Figure 1:**
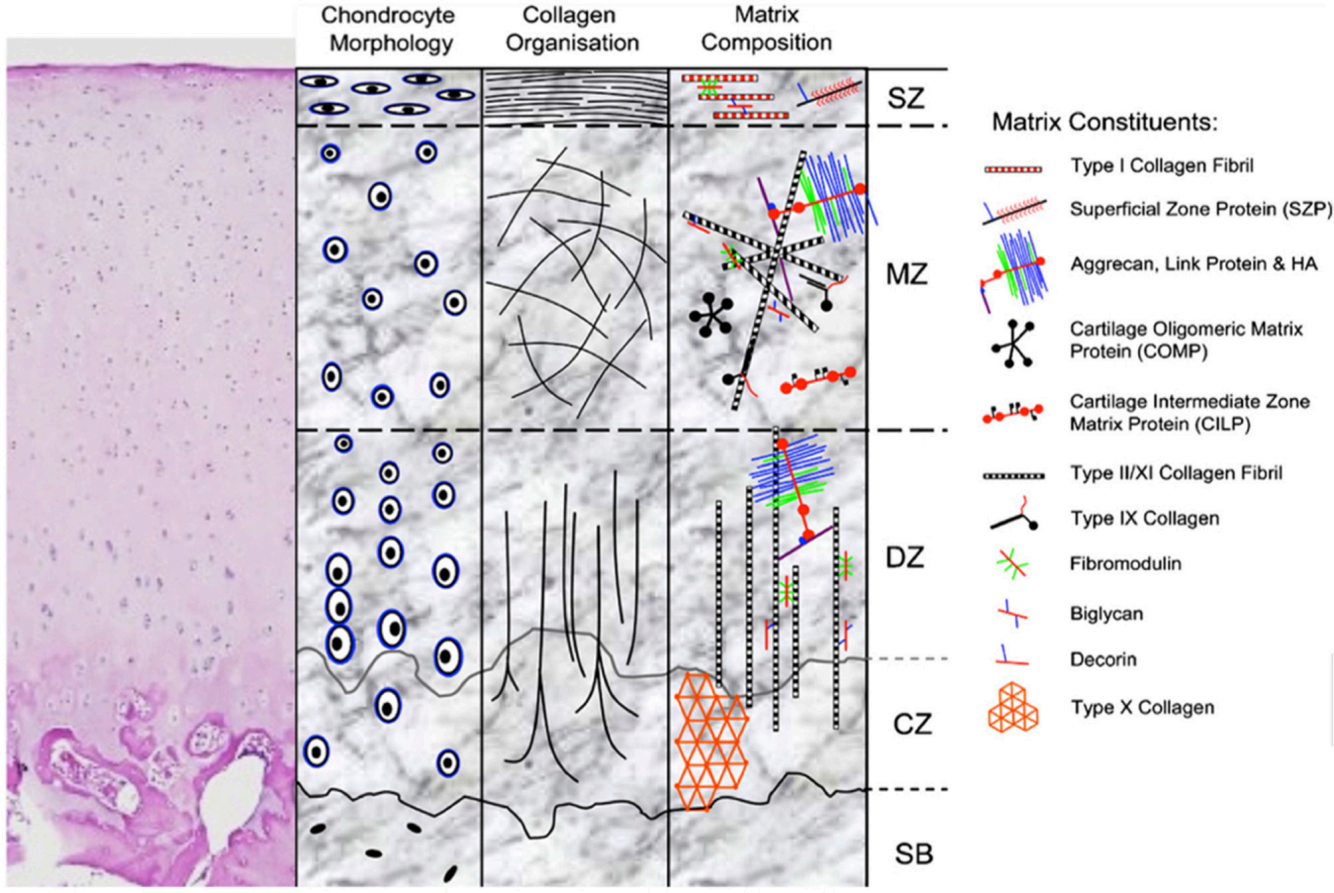
H&E stain and schematic representation of hyaline cartilage morphology and structure. SZ, superficial zone; MZ, middle zone; DZ, deep zone; CZ, calcified zone; SB, subchondral bone. Picture used with permission obtained from J Cytochem Biochem.

Articular cartilage has unique biomechanical properties stemming from its structure (Figure 1) and composition. The lubricated surface provides low friction for articular motion. The extracellular matrix (ECM) and large water content from the effect of proteoglycans provides resistance to strong and repetitive loads of compression and shear. Variations of structure and composition in the different zones allow articular cartilage to resist complex loads and forces encountered in daily activity (4).

Damaged hyaline cartilage is unable to self-repair (5) due to its avascular nature. This characteristic of articular cartilage represents a major challenge in the field of orthopedics. Overtime, either with or without treatment the defect is filled with fibrocartilage, which represents a stiff tissue that doesn't provide the specialized properties of hyaline cartilage (3).

## Current treatment strategies

Surgically many techniques have been developed in an attempt to repair/regenerate cartilage. These can be classified into bone marrow stimulation techniques (drilling, abrasion, and microfracture), direct chondral replacement (mosaicplasty and osteochondral allograft transplantation) and cell culture-based treatment (Autologous Chondrocyte Implantation and Matrix-induced Autologous Chondrocyte Implantation).

Microfracture is the most commonly used technique whilst Autologous Chondrocyte Implantation (ACI) and Matrix-induced Autologous Chondrocyte Implantation (MACI) are used by some surgeons in specific cases. Microfracture is an arthroscopic technique in which holes are created in the subchondral bone to allow blood and bone marrow to proximate, clot, and stimulate cartilage repair (6). ACI involves cartilage being harvested from non/low-weight bearing regions from which chondrocytes are isolated and expanded over 6–8 weeks, after which the cells are implanted back in surgically (7). MACI is a progression of the ACI technique characterized by the delivery of cells in association with a scaffold (8).

Comparison between all three methods have shown no superiority of the newer techniques compared to the less expensive microfractures. A randomized control trial comparing ACI to microfracture showed no significant improvement in cartilage repair outcomes (9), furthermore, no difference was shown comparing ACI to MACI (10). With no significant difference prevailing amongst the three techniques the general surgical inclination has been to opt for microfracture which is a simpler and cheaper option.

Studies involving microfracture show better outcome in patients <40 years old with isolated lesions averaging 2 cm^2^ in size, however at 36 months follow up there was significant reduction in clinical outcome (11). This technique can therefore only be provided to a small subset of patients and lacks long-term outcome data. Reasons for poor long-term outcomes include fibrocartilage production, patient age, functional level, location, and size of the defect (3, 11–13).

## The emergence of cartilage tissue engineering

**Figure 2:**
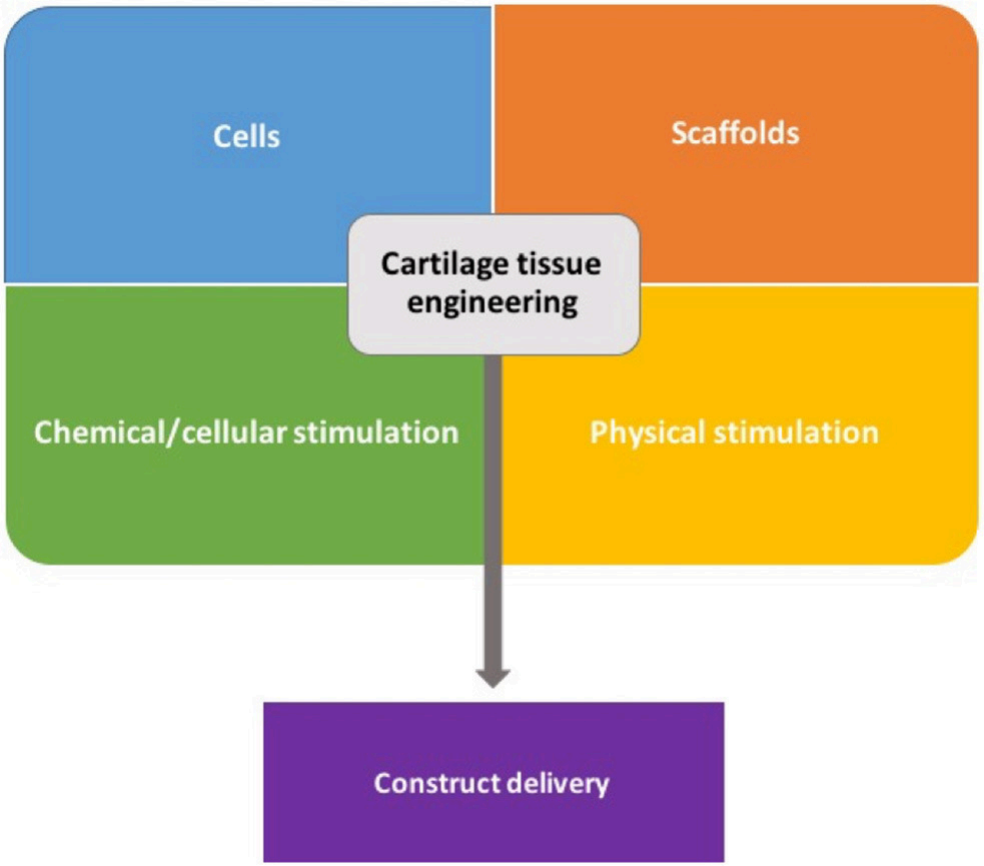
General components used in cartilage tissue engineering.

Poor long-term results and outcomes from bone marrow stimulation techniques like microfracture introduced the field of cartilage tissue engineering (Figure 2). The general principle of tissue engineering involves the use of cells, scaffolds, growth factors, and physical stimulation (diamond concept) to regenerate living tissue (13).

In the field of cartilage, the aim is to develop bio-mimetic tissue that can reliably perform, both biologically and biomechanically, as hyaline cartilage. There is a long history of cartilage tissue engineering attempts with initially engineered components resembling hyaline cartilage, however compared to native cartilage they lacked in mechanical properties (14, 15). Over time there has been additional focus on the biomechanical properties and cell-matrix/scaffold properties. The biomechanical forces being replicated in cartilage engineering include shear, tension, and compression which are endured in day to day activity within native joints (16).

3D bioprinting has emerged as a new technique to overcome some of the difficulties encountered in standard tissue engineering strategies, thanks to its speed, reliability, and precision (17). With respect to cartilage regeneration, the potential superiority of 3D bioprinting is in the ability to provide an efficient and tailored approach in the treatment of unique defect patterns.

Cartilage engineering-based techniques would be indicated in those with pure chondral lesions. If stem cells are used for such a repair approach, then any size of isolated chondral injury can be repaired due to the high replicative ability of these cells, meaning an abundant cell number can be obtained with expansion. With research progression osteochondral defects could theoretically be repaired by building distinct layers with cells differentiated into cartilage or bone.

### Cells–barriers

**Figure 3:**
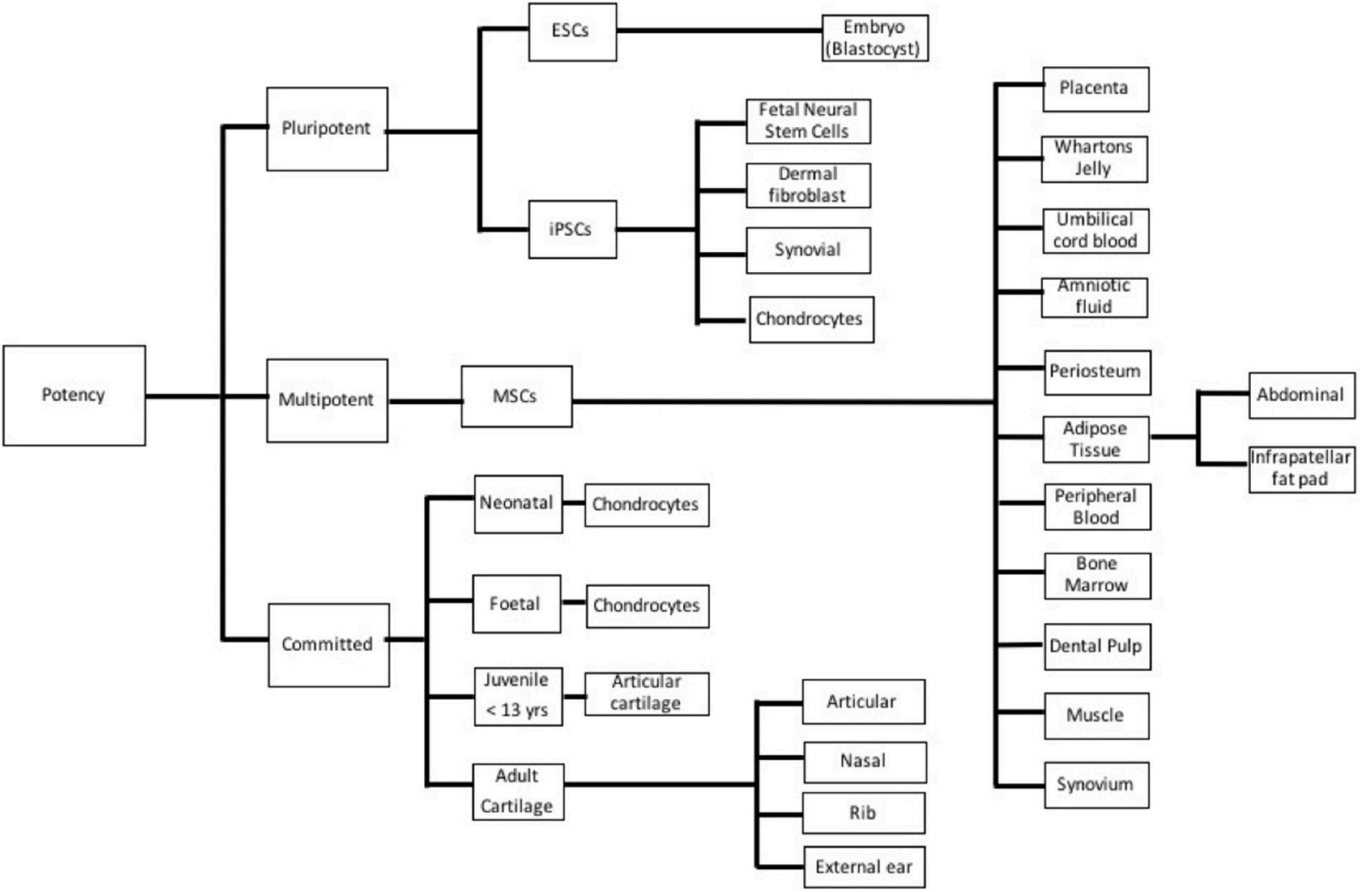
Different cell types used in cartilage tissue engineering based on potency. ESC, Embryonic stem cell; iPSC, Induced pluripotent stem cell; and MSC, Mesenchymal stem cell.

Many different cell lineages of various potencies and sources (Figure 3) have been studied; advantages and disadvantages of the types are outlined in Table 1. Mesenchymal stem cells (MSC) have emerged as a major line with respect to cartilage engineering and show excellent chondrogenic potential (18, 19).

**Table 1:** Advantages and disadvantages of different cell potencies that have been used in cartilage tissue engineering.

**Potency**	**Types**	**Advantages**	**Disadvantages**
Pluripotent stem cells	ESCs	Indefinite self-renewal Multiple cell/tissue lineages	Ethical concerns Tumorigenicity Immune rejection
	IPSCs	Autologous Indefinite self-renewal	Tumorigenicity Cellular reprogramming
Mesenchymal stem cells	–	Autologous Abundant Easily accessible/harvested	Donor site morbidity
Chondrocytes	Fetal	Low immunogenicity	Limited availability
	Neonatal	Low immunogenicity	Limited availability
	Juvenile	Low immunogenicity	Limited availability
	Adult	Autologous	Limited availability Donor site morbidity De-differentiation

*ESC, Embryonic stem cell; IPSC, Induced pluripotent stem cells*.

The optimal tissue sources for harvesting MSCs has traditionally been the bone marrow and adipose tissue, due to easier access and the amount of tissue available. Adipose derived MSCs has been shown to produce superior chondrogenic differentiation when compared to bone marrow derived MSCs (20, 21), furthermore, more MSCs can be isolated per tissue volume when using adipose compared to bone. Abdominal subcutaneous tissue and the infrapatellar fat pad (IFP) are the most commonly used adipose tissue harvest sites for MSC isolation. Although abdominal subcutaneous fat is more abundant, IFP tissue has been shown to be more superior with respect to chondrogenic potential (22).

Irrespective of final cell choice and source there are several key barriers that need to be addressed for clinical translation using cell-based cartilage engineering.

#### Concerns around *in-vitro* lab-based cell expansion

Cell expansion systems are timely and currently require *in-vitro* lab-based cell culture. This opens up concerns with respect to lab sterility, ethics, and risks associated with the use of animal serum-based media products (23–26). Development of a rapid isolation approach may allow avoidance of any *in-vitro* lab-based cell culture if enough cells can be isolated and implanted directly in theater (27, 28), eliminating concerns surrounding sterility and limiting the exposure to animal serum-based media.

Irrespective of isolation timeframes it will be critical to establish protocols for cell culture without animal serum-based media products in view of human clinical translation. The development of animal serum-free culture media using human derived growth supplements for MSC expansion, such as human platelet lysates (29–33) presents a promising solution. Furthermore, development of animal and human serum-free culture media for the expansion of MSC toward clinical applications is another promising strategy (34–36).

#### How many cells are needed based on defect size?

Cell-free cartilage repair techniques such as microfracture are based primarily on lesion thickness (37, 38). Current cell implantation techniques such as ACI and MACI use an expanded number of chondrocytes based on either surgeon preference or rough physiological cartilage cell counts with preference leaning toward higher than normal cell densities (39). The use of higher chondrocyte densities *in-vitro* can inhibit matrix synthesis leading to reduced ECM production (40, 41), whilst using lower densities will not produce enough ECM *in-vitro* (42, 43). Only a few *in-vivo* and *in-vitro* studies report cell densities used and the use of higher cell densities has not been shown to be superior to lower densities, therefore, no optimal clinical cell density has been identified (39).

There is no data on the desired stem cell density in the literature, with research groups tending to use cell counts based on anatomical human data which shows roughly 1 × 10^4^ chondrocytes in 1 mm^3^ of tissue (44). Stem cells have a higher proliferate ability compared to chondrocytes (45), therefore, use of chondrocyte based cell numbers with stem cells will not be an accurate measure of the optimal cell density needed at time of repair. It will be imperative to investigate the optimal cell density per defect volume when using stem cells and taking into consideration other factors such as the matrix type and volumes used.

### Scaffold materials–barriers

The vast majority of cartilage tissue engineering techniques use scaffolds, which help support and create 3D structures of the cellular matrix. Successful scaffold designs optimize certain requirements (46–48) including; biocompatibility, environment, toxicity, degradation rate, pore size, pore geometry, and scaffold stiffness.

Scaffolds can be classified as natural polymers, synthetic polymers, or a hybrid. In cartilage engineering softer polymers, which can initially fill in defects and then solidify at later stages, are the bio-scaffolds that show most promise for clinical application due to their ability to fill complex morphologies which present at the time of surgical repair (49). Soft polymers such as hydrogels have biomimetic properties similar to soft tissue (50), however, at their starting point, i.e., liquid phase, hydrogels have relatively poor mechanical stability, which represents a limitation of their use in this state (51).

#### Can hydrogels provide biomimetic mechanical strength?

Normally hydrogels are printed in a liquid state, which can then solidify after a phase transformation induced by chemical or non-chemical reactions. Cross-linking can be undertaken via photo, thermal or chemical induction (52). This process allows molecules to crosslink and provides increased mechanical stability to the tissue (53, 54).

Over time tunable hydrogel composites have been formulated which can be tweaked to enhance the mechanical properties of the material (55–57) and allow vertical construction of a 3D shape which was another initial limitation of hydrogel use (57). However, each addition or modification that is made can interact and effect the biocompatibility and therefore needs to be thoroughly tested.

#### Can cross-linking of the scaffold be incorporated intraoperatively?

Cross linking can be performed prior to implantation, meaning the engineered construct is made on a bench then implanted separately. This will prevent normal human tissue from being affected directly by cross-linking reactions and the sources used. To date, most *in-vivo* animal models have utilized bench-based formation of cross-linked pre-formed cartilage tissue, with subsequent implantation into simple non-complex defect morphologies which are man-made at time of surgery (58–60).

However, as mentioned earlier implanting pre-formed tissue will be difficult due to the complexity associated with cartilage defect morphology and therefore if at time surgical printing is preferred then the ability to cross link needs to be performed during surgery also leading to the issue of safe intraoperative cross-linking.

A recent pilot study (61) showed an intraoperative direct hand-held repair technique in a sheep model where tissue was formed and cross-linked directly into a defect at time of repair, thus avoiding any pre-printed bench-based tissue engineering techniques. In this study an ultraviolet light was used to cross link the hydrogel material which was preloaded with a photo initiating material, the light source was used as a separate component to the hand-held bioprinter. Macroscopically the use of the light source didn't affect surrounding healthy tissue. This study also used simple man-made defects and therefore doesn't confirm the ability to directly bio-print engineered cartilage into complex morphologies, however, if validated with more complex lesion patterns provides a more suitable option compared to implanting pre-formed cartilage tissue.

In future studies it will be ideal to directly add such a source to a hand-held printing device limiting the need for additional components and complexity to the surgical approach.

### Chondrogenic stimulation–barriers

Molecules including growth factors and soluble non-protein chemical compounds chemically regulate cell behavior. Molecules bind to surface receptors on stem cells to activate intracellular signal pathways controlling cellular proliferation, differentiation, and synthesis. Mesenchymal stem cells have an important paracrine effect on cartilage formation and promoting an anti-inflammatory environment (62, 63).

Many combinations such as transforming growth factor (TGF) beta-3 with bone morphogenetic protein (BMP) 2 have induced chondrogenesis in adult stem cell use (64). Irrespective of the combination used, during *in-vitro* experimentation chemical stimulants can be provided (via culture media) periodically, producing consistent chondrogenic differentiation.

However, regular delivery and monitoring of stimulation can't be translated into an *in-vivo* setting as the native knee synovium must take over nutritional control. The native synovium in an adult is not catered to newly growing cartilage, meaning the desired growth factors and respective concentrations provided in *in-vitro* studies will not be present *in-vivo*, therefore, synovial take-over of nutritional regulation becomes an unknown obstacle in the way of human clinical trials.

#### Can engineered cartilage tissue be maintained in the body in the long term?

With a view of translating tissue engineered cartilage into the human knee, a major barrier will be with the formation of reliable and consistent chondrogenic tissue over time. Human blood supply will have growth factors and chemical stimulants that can assist with chondrogenic stimulation, however, highest activity is during fetal/young stages of life with increasing age associated with less stimulation and organization of chondrocytes and extracellular matrix (65). Therefore, theoretically if engineered tissue (composed of newly forming cartilage cells from a stem cell base) is used in adolescent cartilage repair we have no certainty of cellular control and regulation *in-vivo*.

Short term results are promising with respect to the use of cartilage tissue engineering methods in an *in-vivo* animal model (58, 59, 61). However, no mid/long-term (>12 weeks) *in-vivo* animal studies using cartilage tissue engineering and 3D bioprinting methods have been performed, therefore, no superiority compared to current clinical techniques such as microfracture and ACI can be ascertained. Mid/long-term *in-vivo* animal studies using tissue engineering techniques will be crucial in validating the ability of the native synovium in nourishing and maintaining engineered cartilage over large periods of time, this data will be pivotal to obtain prior to any progression to human clinical trials.

### Physical/environmental stimulation–barriers

With respect to mechanical and environmental stimulation, if tissue is to be printed directly *in-vivo* then native forces encountered by the knee in conjunction with synovial nourishment should directly take over adequate stimulation. Therefore, with an approach avoiding lab-based tissue growth there is no major concern with the ability to mimic the environment of the human knee.

The replication and delivery of such an environment is only important when trying to maintain a bench based *in-vitro* model of repair research where a bio-printed piece of tissue is maintained in a lab for *in-vitro* investigation or for *in-vivo* models using pre-formed tissue which is maintained in a lab then implanted at a later date. In these studies, tissue will need to be maintained as close to the native *in-vivo* environment as possible whilst in the lab. Therefore, ideally these cartilage constructs should be exposed to the different types of mechanical forces encountered by the native knee, such as shear stress, perfusion, hydrostatic pressure, and compression (16, 66).

#### Recreating the properties of the human knee in a 3D culture environment–3D bioreactors

Well-designed bioreactor systems can provide stimulation in one consistent and regular setting therefore providing a culture environment that promotes cartilage growth (67).

##### Single stimulation bioreactor environments

Induction of shear force to promote cartilage growth has been demonstrated using rotating vessels (68–70), spinner flasks (71, 72), and stirred double chambers (73). Induction of perfusion to promote cartilage growth has been demonstrated using fluid perfusion bioreactor systems either using a unidirectional (74) or a bidirectional (75) bioreactor. Induction of hydrostatic pressure to promote cartilage growth has been demonstrated using fluid filled chambers with water pumps (76, 77). Induction of compression to promote cartilage growth has been widely demonstrated (78).

##### Bioreactors for inducing multiple/combined mechanical force

Bioreactors applying multiple mechanical properties are currently being developed. One study combining intermittent unconfined shear and compressive loading for 2.5 weeks showed increase in glycosaminoglycan and collagen type II production in human chondrocytes by 5.3- and 10-fold, respectively, after simultaneous stimulation (74).

More development is warranted into the application of multiple mechanical forces within bioreactor systems with respect to *in-vitro* cartilage tissue engineering.

### Construct delivery—barriers

Modern advancement in 3D bioprinting technology opens up novel techniques with respect to the delivery of engineered human tissue. As mentioned earlier with respect to cartilage tissue engineering the ideal materials for printing are hydrogels given their ability to accommodate complex morphologies (49). With respect to 3D bioprinting of engineered cartilage tissue the ideal printing device would need to be small, portable, and operator friendly to allow for direct intraoperative printing into defects.

**Figure 4:**
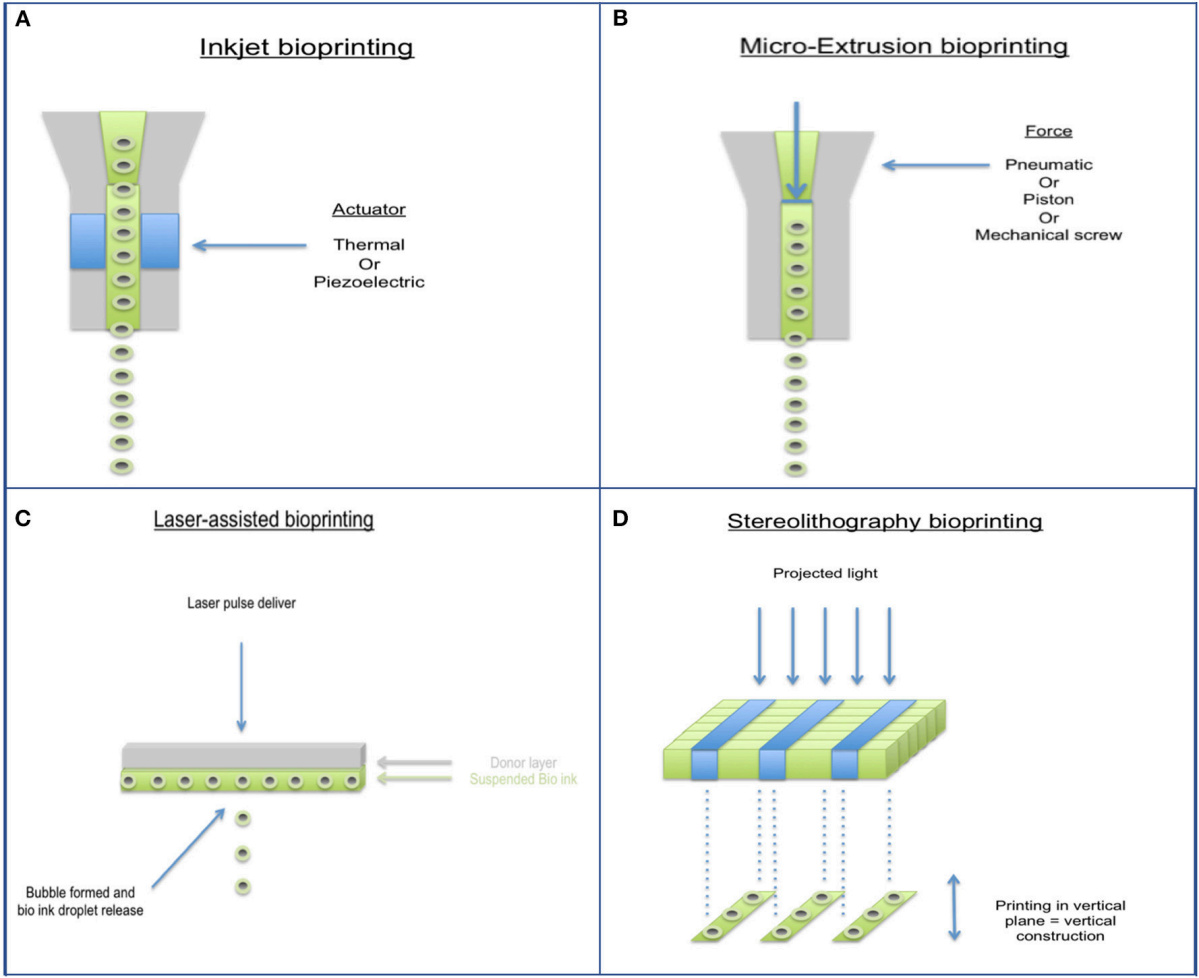
Different types of 3D Bioprinting methods. **(A)** Inkjet bioprinter, **(B)** Micro-extrusion bioprinter, **(C)** Laser-assisted bioprinter, and **(D)** Stereolithography bioprinter.

Bioprinting has largely come about to combat the limitations of past tissue engineering methods and allows for accurate control of cell distribution, production of biomimetic geometry, and rapid production of construct (79, 80). With respect to cartilage tissue repair, it is impossible to accurately characterize defects even with current advances in medical imaging, therefore bioprinting techniques in this field need to be focused more on flexibility rather than computer aided precision. Several different types of bioprinters have been developed (Figure 4), characteristics and disadvantages of each type are shown in Table 2. Hand-held bioprinting used in conjunction with hydrogels are best suited to achieve this as they can plug defects of any nature easily like using a glue stick/gel, avoiding the need for large and heavy machinery in the operating theater (81).

**Table 2:** Characteristics and disadvantages of the different 3D Bioprinting methods.

**Bioprinter type**	**Cell viability %**	**Resolution**	**Speed**	**Disadvantages**
Inkjet	80–90	High	Fast	Clogging of head Settling effect of bio ink
Micro extrusion	<80	Moderate	Slow	Large mechanical stressors
Laser assisted	>90	High	Medium	Expensive Immature field Unknown effects of laser on cells
Sterolithography	>85	High	Fast	Limited compatibility with liquid/gel biomaterials

**Figure 5:**
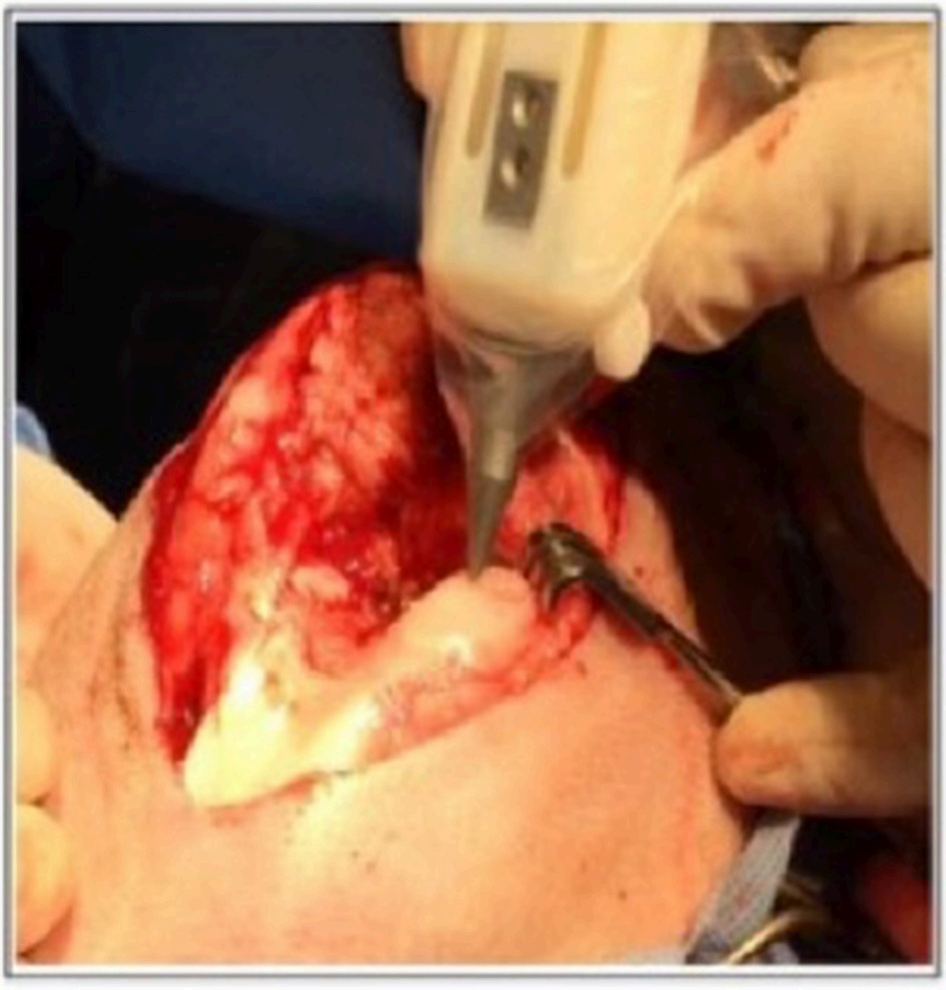
Intra-operative photograph of the Biopen in action in a sheep model. [Modified from and used with permission from Di Bella et al. (81), under CC BL].

**Figure 6:**
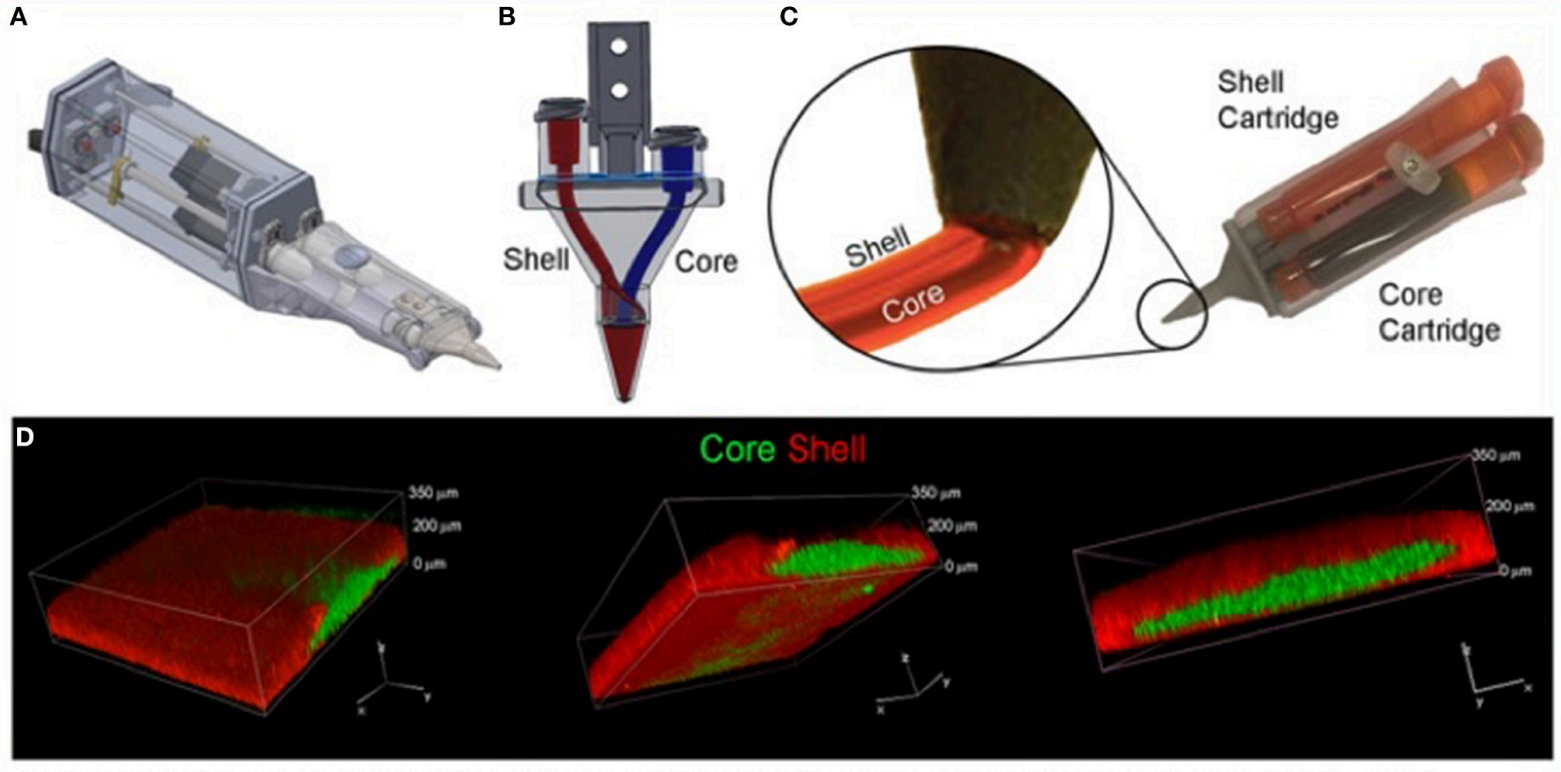
Core/Shell 3D printing by co-axial extrusion. **(A)** Schematic representation of the 3D co-axial handheld printer. **(B)** Schematic representation of the co-axial nozzle. **(C)** Picture of the cartridges dedicated to Core and Shell loading in the printer, with relative magnification of the nozzle during co-axial deposition. **(D)** Representative 3D rendered confocal images of Core/Shell printed sample labeled with fluorescent beads. The hydrogel shell is shown in red channel, while the cellular/hydrogel core is shown in green channel. The panel shows the same image representative of 3D rendered of superimposed green and red channels in three different orientations. [Modified from and used with permission from Duchi et al. (83) under CC BL].

The most suitable mechanism to fit a portable hand-held type device would be extrusion printing given the compactable nature of the mechanism, an example of this is the “bio pen” which uses pneumatic extrusion-based technology to allow printing of hydrogels (82), Figure 5 shows the intraoperative use of the bio pen in an sheep model. This device can be loaded with two cartridges allowing for a co-axial based printing method (Figures 6A–D) which can concentrate cells in the middle core with an outer hydrogel shell layer that can be crosslinked and protect the inner cellular tissue (83).

Engineered tissue using a portable hand-held device can be printed directly during surgery on the spot with the top of the construct being immediately crosslinked using methods mentioned earlier. This approach allows for implant stability within the lesion (61). However, when using non-portable printers and non-hydrogel-based scaffolds/materials the formed implant will need to be manually positioned (requires precise geometry when initially created) into the lesion and then anchored with either sutures or a flap approach.

*In-vivo* animal delivery of engineered cartilage tissue has been performed to-date using large incisions over the knee, similar to those made in conventional knee replacement surgeries (61, 84). Larger incisions, however, increase the risk of wound complications (e.g., infection and wound dehiscence) and reduce cosmetic satisfaction, both of which are important factors to consider in treatment of a young-middle aged population.

#### Can engineered cartilage tissue be provided using less risk adverse minimally invasive techniques?

Arthroscopic knee surgery (85) (key-hole surgery) can provide such a solution and is performed in a day surgery setting allowing patients to return to daily activity almost immediately. Technically speaking the desired bioprinting device would therefore be incorporated as an attachment into an arthroscopic kit, once again the most suited to this would be a small hand-held bioprinter. This microsurgical kit/device will need adequate valdiation and training prior to use.

**Figure 7:**
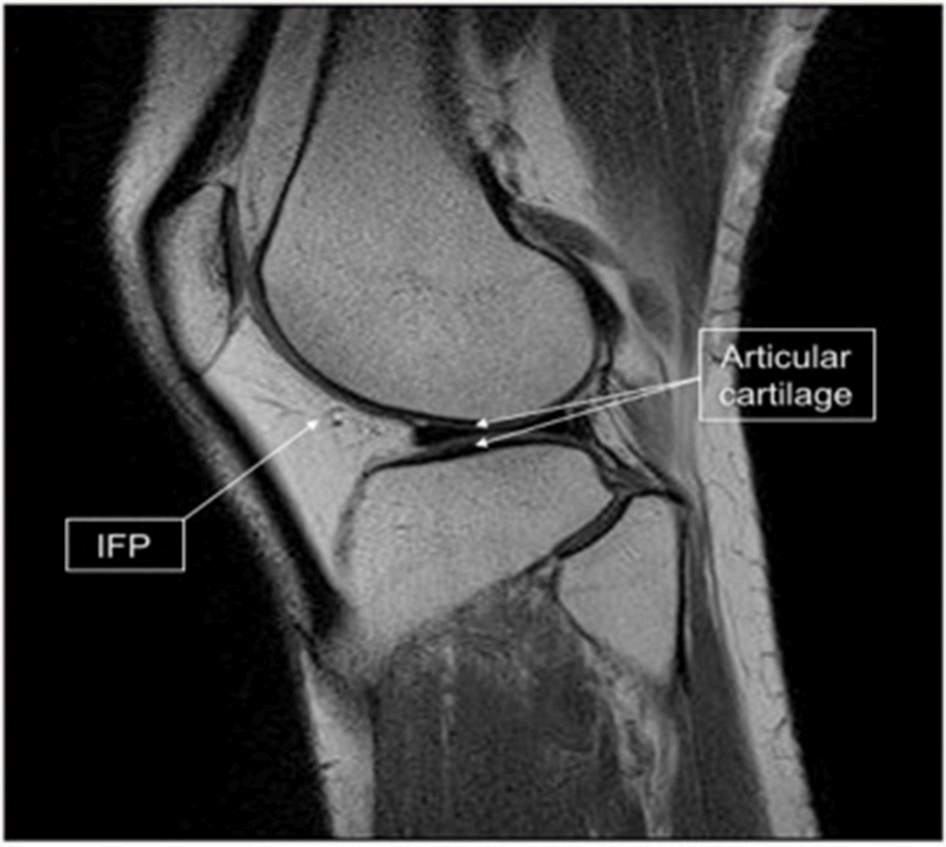
Infrapatellar fat pad (IFP) location and harvested tissue. Sagittal magnetic resonance imaging scan of the knee showing the relationship of the IFP (arrow) to the articular cartilage (double arrow). [Modified from and used with permission from Hindle et al. (22) under CC BL].

If as mentioned earlier a rapid stem cell isolation can be established to fit within a surgical time frame (28) then this will open up the ability to harvest tissue, isolate stem cells then directly re-implant a cell/scaffold interface into knee joint in one single operation. Furthermore, if stem cells can be harvested from a source like the infrapatellar fat pad (IFP) (86) which is in close proximity to the knee joint (Figure 7) the operation can be performed using one surgical site. Arthroscopic harvest of the IFP for adipose-derived mesenchymal stem cells isolation has been performed successfully in a rabbit model (22).

#### Will the delivered construct integrate effectively with host tissue?

**Figure 8:**
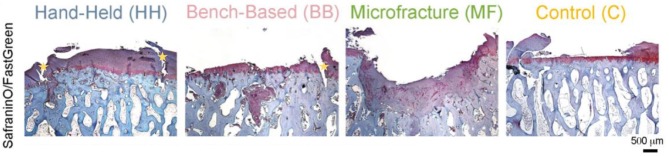
Histology (Safranin/Fast green staining) showing better new cartilage formation in the HH group compared to the other groups. Subchondral cysts and collapse are seen in the BB and MF group, with fibrocartilage formation. Voids at the implanted/native tissue interfaces are seen in the HH and BB groups indicated by the yellow stars. HH, Hand held Biopen printed scaffold; BB, Bench Based 3D printed scaffold; MF, Microfractures; C, Negative Control (defect left empty). Voids at the implanted/native tissue interfaces are seen in the HH and BB groups. [Modified from and used with permission from Di Bella et al. (81), under CC BL].

Another uncertainty is the ability of engineered cartilage tissue to integrate with healthy tissue. It is evident that superior cartilage repair can be achieved using a hand-held printing method, however both hand-held and bench-based printing methods can produce the development of voids (Figure 8) between the edges of the implanted tissue and health native tissue (61).

A possible solution to overcome this would be to apply an initial coat of biocompatible adhesive (87) into the defect prior to implantation allowing better proximation of tissue. Subsequently it will be pivotal to investigate if such a method then allows interaction of the implanted tissue with the healthy tissue, this could be investigated adding tracking materials/dyes to the constructed tissue, and then assessing movement/integration at different time points (88, 89).

## Conclusion–human clinical translation

Cartilage repair techniques using stem cells and 3D bioprinting technology still remains elusive with respect to human clinical translation. Many challenges remain to be addressed and overcome as described in this review. More focus on *in-vivo* animal studies will be essential to investigate many of these barriers. Whilst continued progress is made on such issues it is important to begin setting up larger scale *in-vivo* animal trials which will be the next step toward human clinical translation.

## Author contributions

SF completed a literature search, contributed to the preparation of the manuscript, and critically revised the manuscript. CD, GW, and PC contributed ideas, content to the manuscript, and critically revised the manuscript.

### Conflict of interest statement

The authors declare that the research was conducted in the absence of any commercial or financial relationships that could be construed as a potential conflict of interest.

## Abbreviations

3D: Three dimensional
ACI: Autologous Chondrocyte Implantation
MACI: Matrix-induced Autologous Chondrocyte Implantation
MSC: Mesenchymal Stem Cell
TGF: Transforming Growth Factor
BMP: Bone Morphogenetic Protein.
